# *GRK6* Depletion Induces HIF Activity in Lung Adenocarcinoma

**DOI:** 10.3389/fonc.2021.654812

**Published:** 2021-05-31

**Authors:** Sumei Yao, Ayse Ertay, Yilu Zhou, Liudi Yao, Charlotte Hill, Jinliang Chen, Yangbo Guan, Hui Sun, Rob M. Ewing, Yifei Liu, Xuedong Lv, Yihua Wang

**Affiliations:** ^1^ Department of Respiratory Medicine, The Second Affiliated Hospital of Nantong University, Nantong, China; ^2^ Biological Sciences, Faculty of Environmental and Life Sciences, University of Southampton, Southampton, United Kingdom; ^3^ Institute for Life Sciences, University of Southampton, Southampton, United Kingdom; ^4^ Department of Urology, Affiliated Hospital of Nantong University, Nantong, China; ^5^ Department of Pathology, Affiliated Hospital of Nantong University, Nantong, China; ^6^ Medical School of Nantong University, Nantong, China

**Keywords:** GRK6, HIF, lung adenocarcinoma, hypoxia, EMT

## Abstract

G protein-coupled receptor kinase 6 (GRK6) is expressed in various tissues and is involved in the development of several diseases including lung cancer. We previously reported that GRK6 is down-regulated in lung adenocarcinoma patients, which induces cell invasion and metastasis. However, further understanding of the role of GRK6 in lung adenocarcinoma is required. Here we explored the functional consequence of *GRK6* inhibition in lung epithelial cells. Analysis of TCGA data was coupled with RNA sequencing (RNA-seq) in alveolar epithelial type II (ATII) cells following depletion of *GRK6* with RNA interference (RNAi). Findings were validated in ATII cells followed by tissue microarray analysis. Pathway analysis suggested that one of the Hallmark pathways enriched upon GRK6 inhibition is ‘Hallmark_Hypoxia’ (FDR = 0.014). We demonstrated that *GRK6* depletion induces HIF1α (hypoxia-inducible factor 1 alpha) levels and activity in ATII cells. The findings were further confirmed in lung adenocarcinoma samples, in which GRK6 expression levels negatively and positively correlate with HIF1α expression (*P* = 0.015) and VHL expression (*P* < 0.0001), respectively. Mechanistically, we showed the impact of GRK6 on HIF activity could be achieved *via* regulation of VHL levels. Taken together, targeting the HIF pathway may provide new strategies for therapy in GRK6-depleted lung adenocarcinoma patients.

## Introduction

G protein-coupled receptor kinases (GRKs) are a family of kinases that play a critical role in G protein-coupled receptors (GPCRs) homologous desensitization. GRKs phosphorylate specific serine and threonine residues of activated GPCRs which promote high affinity binding of arrestins and then suppress further G protein activation by interrupting receptor-G protein coupling (1–3). Desensitization of GPCRs has a critical role in maintaining homeostasis. As such, abnormal GPCRs desensitization can cause a variety of human diseases, including autoimmune diseases (4), asthma (5), heart failure (6), Parkinson’s disease (7), inappropriate diuresis (8) and tumour progression and metastasis (9). Therefore, GRKs are important therapeutic targets for these diseases.

G protein-coupled receptor kinase 6 (GRK6) is a member of the GRK family, which is expressed in various tissues and involved in the development of several diseases (10–12). High expression of GRK6 has been reported in hepatocellular carcinoma (13), colorectal cancer (14); whilst lower expression was reported in hypopharyngeal squamous cell carcinoma (15) compared to normal tissues. Further, *Grk6* knock out mice (*Grk6* ^-/-^) showed a significant increase in the growth and metastasis of Lewis lung cancer (LLC) compared to the control mice (*Grk6* ^+^/^+^) (2). Our previous study suggested that GRK6 expression was significantly down-regulated in lung adenocarcinoma patients, and its level was an independent prognostic factor for overall survival (16). Moreover, we also showed that the promoter region of the *GRK6* gene was hyper-methylated in lung adenocarcinoma tissues compared to the normal tissue samples, leading to a down-regulation of *GRK6* expression and in turn, inducing cell invasion and metastasis (17). However, further understanding of the role of GRK6 in lung adenocarcinoma is required.

In this study, we aimed to investigate the functional consequence of *GRK6* depletion in lung epithelial cells. Analysis of TCGA data was coupled with RNA sequencing (RNA-seq) in alveolar epithelial type II (ATII) cells following the depletion of *GRK6* with RNA interference (RNAi). Tissue microarrays were used to investigate the expression and function of *GRK6* in lung adenocarcinoma. Our data suggests that *GRK6* depletion induces HIF1α (hypoxia-inducible factor 1 alpha) activity. Targeting the HIF pathway may provide new strategies for therapy in GRK6-depleted lung adenocarcinoma patients.

## Materials and Methods

### Cell Culture, Transfections, and Reagents

ATII (alveolar epithelial type II, kindly provided by Prof Julian Downward, The Francis Crick Institute, UK) cells (18–21) were cultured in DCCM-1 (Biological Industries Ltd) supplemented with 10% new-born calf serum (NBCS) (Life Technologies), 1% penicillin, 1% streptomycin, and 1% L-glutamine (all from Life Technologies). All cells were kept at 37°C and 5% CO_2_. No mycoplasma contamination was detected in the cell lines used.

Short interfering RNA (siRNA) oligos against *GRK6* or control siRNA were purchased from Biomics Biotechnologies Co., Ltd, China. Sequences are available from an earlier publication (17). Cells were transfected with the indicated siRNA oligos at a final concentration of 35 nM using Dharmafect 2 reagent (Dharmacon).

### RNA Isolation, Library Construction, and Sequencing

To identify global transcriptomic changes in ATII cells upon *GRK6* depletion, RNA sequencing (RNA-seq) was performed. In brief, ATII cells were transfected with either control siRNA or siRNA against *GRK6* for 3 days. Total RNA was isolated using an RNeasy mini kit (Qiagen) according to the manufacturer’s instructions and quantified using a Nanodrop Spectrophotometer 2000c (Thermo Fisher Scientific). A total amount of 3 µg RNA per sample was used as input material for library construction. Sequencing libraries were generated using NEBNext^®^ UltraTM RNA Library Prep Kit for Illumina^®^ (NEB, Ipswich, Massachusetts, USA) following the manufacturer’s instructions. Libraries were pooled in equimolar and sequenced using the paired-end strategy (2 × 150) on the Illumina NovaSeq 6000 platform following the standard protocols (Novogene, UK). RNA-seq data have been deposited in the Gene Expression Omnibus (GEO) database (accession code GSE164921).

### RNA-seq Data Analysis

Quality control of RNA-seq data was performed using FastQC (http://www.bioinformatics.babraham.ac.uk/projects/fastqc) and MultiQC (22). Trim Galore (https://github.com/FelixKrueger/TrimGalore) was used to trim adapters, reads with low quality (< 30), and short length (< 50 bp). RNA-seq reads were mapped to Human genome Ensembl GRCh38 using Hisat2 (23) (version 2.1.0) with default codes. Sam files were transformed into bam files using samtools (24) (version 1.9). The read counts of each gene were summarized using featureCounts (25) (version 1.6.5). Raw read counts were imported into RStudio (version 3.6.1) and analysed by using R package of DESeq2 (26) (version 1.26.0). Transcripts with low abundance (under 10 counts across all samples) were removed. Genes with a false discovery rate (FDR) *P*-value less than 0.05 adjusted by using Benjamini–Hochberg (BH) method (or q-value) were considered as differentially expressed genes (DEGs). Gene ontology (GO) enrichment analysis was generated through ToppGene (ToppGene Suite for gene list enrichment analysis and candidate gene prioritization) website (https://toppgene.cchmc.org/). Parameter was set with a FDR < 0.05. All downstream analysis was performed in RStudio (version 3.4.4).

### Data Mining *GRK6* Related Data From the Cancer Genome Atlas (TCGA)

The expression of mRNAs in the TCGA lung adenocarcinoma (LUAD) (IlluminaHiSeq) dataset was obtained from the UCSC Xena Browser (https://xenabrowser.net/). To separate the low and high *GRK6* group in the TCGA dataset, hierarchical cluster was performed on the high correlated genes with GRK6 *via* Pearson analysis in RStudio (version 3.4.4). According to the correlation analysis, there were 17 samples in the high *GRK6* group and 26 samples in the low *GRK6* group. Then, an unpaired *t-test* was performed to identify significantly expressed mRNAs (FDR < 0.05) between the high and low *GRK6* groups in RStudio (version 3.4.4). Codes are available upon request.

### Identification of Top Hit Genes and Pathway Analysis

The statistically significant (FDR < 0.05) differentially expressed mRNAs in the TCGA (IlluminaHiseq) dataset that were highly expressed in the low GRK6 lung adenocarcinoma group were merged with statistically different genes in the RNA-sequencing dataset, which showed higher gene expression in si*GRK6* samples compared to the control samples by using RStudio (version 3.4.4) to identify the top hit candidate gene(s) (**Figure 2**).

For pathway analysis, Metascape (https://metascape.org/gp/index.html#/main/step1) was used to detect functional enrichment of the identified top hit genes. The pathways were sorted from the lowest q-value and pathways with a q-value of less than 0.05 were chosen to create a histogram plot in GraphPad Prism 8.

### Western Blot Analysis

Western blot analysis was performed with lysates from cells lysed with urea buffer (8M urea, 1M thiourea, 0.5% CHAPS, 50 mM DTT and 24 mM spermine). The bound proteins were separated on SDS polyacrylamide gels and subjected to immunoblotting with the indicated antibodies. Primary antibodies were from Proteintech (GRK6, Catalog No. 11439-1-AP, 1:1000) BD Transduction Laboratories™ (HIF1α, Catalog No. 610958, 1:1000) and Cell Signalling Technology (β-tubulin, Catalog No. 86298, 1:5000). Signals were detected using an Odyssey imaging system (LI-COR) or an ECL detection system (GE Healthcare, Chicago, IL, USA), and evaluated using ImageJ (version1.42q) software (National Institutes of Health) (Bethesda, MD, USA).

### qRT-PCR

Real-time quantitative RT-PCR was performed using gene-specific primers (QuantiTect Primer Assays, Qiagen) for *CA9* (QT00011697), *NDRG1* (QT00059990) or *ACTB* (β-actin) (QT01680476) with QuantiNova SYBR Green RT-PCR kits (Qiagen). Relative transcript levels of target genes were normalised to *ACTB* (β-actin).

### Clinical Data and Tissue Samples

The study population comprised of 174 lung adenocarcinoma (LUAD) patients who were examined and treated at the Thoracic Surgery Department of the Affiliated Hospital of Nantong University and Thoracic Surgery Department of Second Affiliated Hospital of Nantong University between January 1, 2015, and December 31, 2016. The median age of patients at the time of diagnosis was 63 years (range 41–83 years). Study protocol was approved by the Ethics Committee of the Affiliated Hospital of Nantong University (No. 2018-L068), and all experiments were performed in accordance with approved guidelines of the Affiliated Hospital of Nantong University. Written informed consent was obtained from the patients for publication of this study and any accompanying images. Details of the clinical and demographic information were collected retrospectively. All patients underwent standard surgery aiming for maximal tumour resection. Patient clinical data were recorded in detail, and the diagnoses were confirmed by at least two pathologists. Tumour histological grades and clinical stages were evaluated according to the pathological results after surgery. All tumours were staged according to the pathological tumour/node/metastasis (pTNM) classification (7th edition) of the International Union against Cancer.

### Tissue Microarray (TMA) Construction and Immunohistochemistry Analysis (IHC)

Tissue microarray system (Quick-Ray, UT06, UNITMA, Korea) in the Department of Clinical Pathology, Nantong University Hospital, Jiangsu, China, was used to generate TMA. Specifically, core tissue biopsies (2 mm in diameter) were taken from individual FFPE blocks and arranged in recipient paraffin blocks. TMA specimens were cut into 4 µm sections and placed on super frost-charged glass microscope slides. TMA analysis was used as a quality control for hematoxylin and eosin staining. Tissue sections were deparaffinized and rehydrated through graded ethanol. Antigen retrieval was performed with 0.01 M citrate buffer pH 6.0 and microwave heat induction. Endogenous peroxidase activity was blocked with 3% H_2_O_2_ for 30 min. Sections were then incubated with a rabbit polyclonal antibody specific to GRK6 (1:100; Proteintech, 11439-1-AP), HIF1α (1:100; Proteintech, 20960-1-AP) and VHL (1:100; Abcam, ab140989) at 4°C overnight, followed by incubation with a biotinylated anti-rabbit secondary antibody at 37°C for 30 min. Slides were then processed using horseradish peroxidase and 3,3-diaminobenzidine chromogen solution and counterstained with hematoxylin. The staining intensity of GRK6, HIF1α or VHL for each slide was evaluated and scored by two independent pathologists. Staining intensity was scored as follows: 0 (negative), 1+ (weak staining), 2+ (moderate staining), and 3+ (intense staining). For each of the four staining intensity scores, the percentage of cells stained at each intensity were determined. The intensity percentage score was the product of staining intensity and percentage of stained cells. The final staining scores were then evaluated from the sum of the four intensity percentage scores; thus, the staining score had a range from the minimum value of 0 (no staining) to a maximum of 300 (100% of cells with 3+ staining intensity), as described previously (27). The cut-off of 140 was selected to evaluate expression: score 0–140 was considered low expression, while 141–300 was considered high expression. For all subsequent analyses, GRK6, HIF1α and VHL protein expression levels were considered either as “low” or “high” according to these cut-off values.

### Statistical Analysis

Two-tailed, unpaired Student’s *t-test* for the TCGA data were performed in RStudio (version 3.4.4). For multiple *t*-test, P-values were adjusted by using Benjamini-Hochberg (BH) method. Codes are available upon request. Fisher’s exact test was used to evaluate the relationship of GRK6 and HIF1α expression in lung adenocarcinoma patient samples in IHC using GraphPad Prism 8 software. *P* < 0.05 was considered statistically significant.

## Results

### Global Transcriptomic Changes in ATII Cells Upon *GRK6* Depletion

We previously reported that *GRK6* knockdown promotes cell migration and invasion in lung epithelial cells (17). To determine if, and how, lung epithelial cells responded to *GRK6* inhibition, we characterised the global transcriptomic changes in alveolar epithelial type II (ATII) cells transfected with either siRNAs against *GRK6* (si*GRK6*) or control siRNA (Control) by performing RNA sequencing (RNA-seq). Principal component analysis (PCA) showed good separation between Control compared to si*GRK6* samples (n = 3 in each group) (**Supplementary Figure 1**).

**Figure 1:**
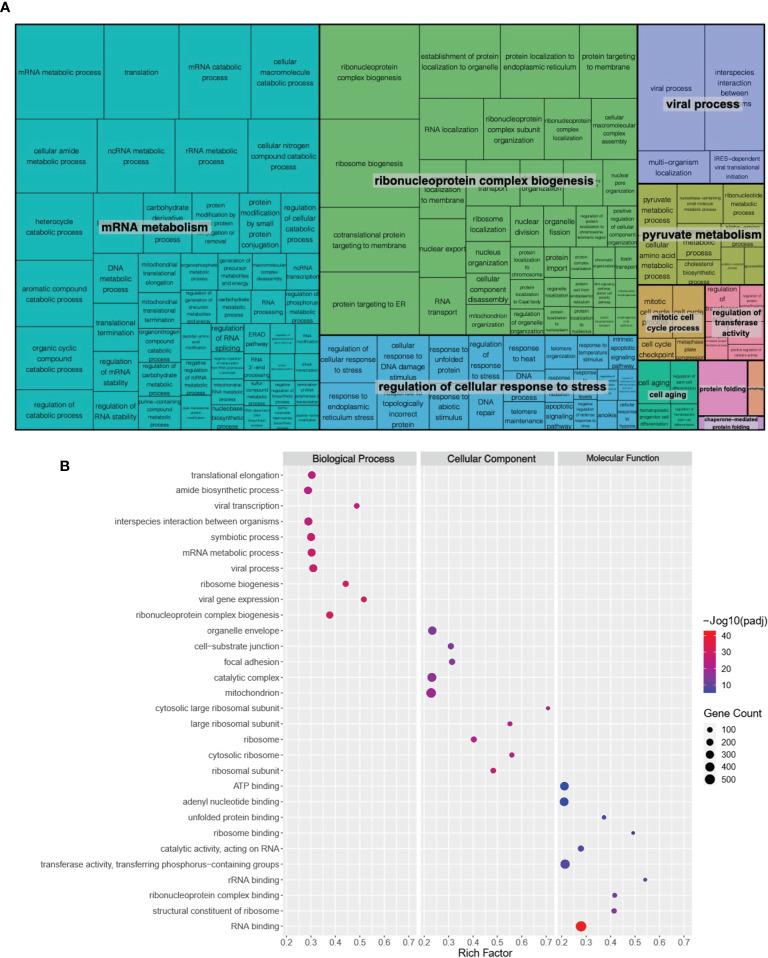
Global transcriptomic changes in ATII cells upon *GRK6* depletion. **(A)** REVIGO TreeMap showing Gene Ontology (GO) analysis of upregulated differentially expressed genes (DEGs) in ATII cells transfected with siRNAs against *GRK6 vs.* control siRNA. Common colours represent groupings based on parent GO terms, and each rectangle is proportional to the relative enrichment of the GO term compared to the whole genome. Genes with false discovery rate (FDR) < 0.05 were considered as DEGs. **(B)** Scatter plot showing the top 10 enriched GO terms from 3 categories (biological process, cellular component, and molecular function) according to rich factors. Rich factor is the percentage of DEGs enriched gene count in the given annotated GO terms. The sizes of circles represent gene counts, and the colours of circles represent the -Log_10_ of the adjusted *P*−values (padj). Values less than 0.05 were considered as statistically significant.

Genes with a false discovery rate (FDR) adjusted *P* value (or q-value) of less than 0.05 were considered as differentially expressed genes (DEGs). In total, 7,116 DEGs were identified, including 3,430 up-regulated (**Supplementary Table 1**) and 3,686 down-regulated (**Supplementary Table 2**). We then performed gene ontology (GO) enrichment analysis of the identified DEGs using ToppGene (ToppGene Suite for gene list enrichment analysis and candidate gene prioritization) website (https://toppgene.cchmc.org/). The results were grouped into molecular function (MF), biological process (BP), and cellular component (CC). Interestingly, several disease-related pathological terms were identified, including mRNA metabolism, ribonucleoprotein complex biogenesis, and regulation of cellular response to stress (FDR < 0.05; **Figures 1A, B**; **Supplementary Tables 3** and **4**).

### Candidate Pathways Enriched Upon *GRK6* Inhibition Are Identified by TCGA Analysis Coupled With RNA-seq

**Figure 2:**
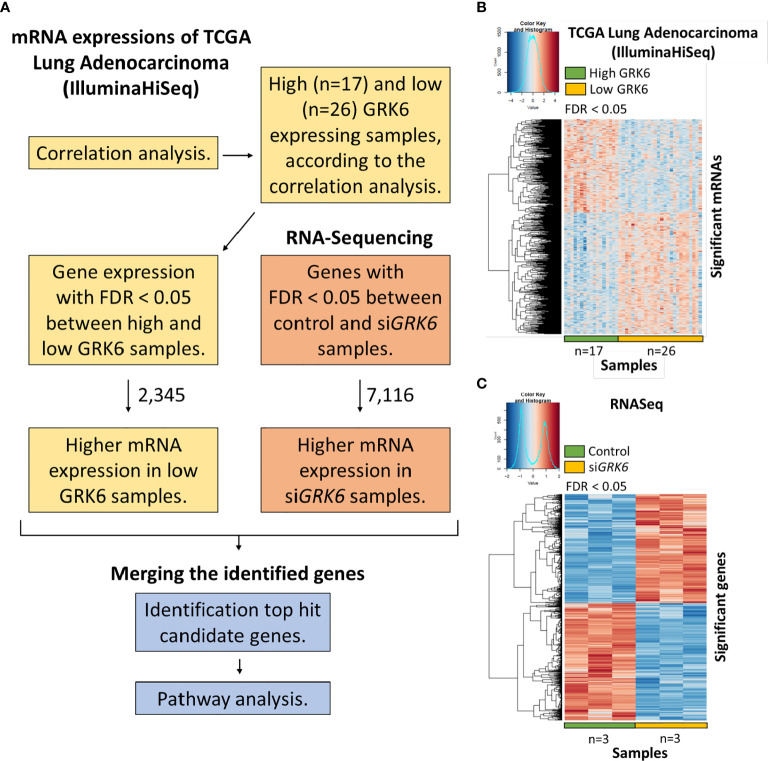
The analysis to identify candidate genes upon *GRK6* inhibition. **(A)** In brief, TCGA analysis coupled to RNA sequencing in ATII cells upon *GRK6* depletion (si*GRK6*) was used (details in Methods). FDR: false discovery rate. **(B)** Heat-map showing DEGs (differentially expressed genes) between low *GRK6* (n = 26) and high *GRK6* (n = 17) expressing lung adenocarcinoma samples from TCGA analysis. Red indicates up-regulation and blue indicates down-regulation. Genes with false discovery rate (FDR) adjusted P-values less than 0.05 were considered as DEGs. P-values were adjusted by using Benjamini-Hochberg (BH) method. **(C)** Heat-map showing DEGs in ATII cells transfected with siRNA against *GRK6* (si*GRK6*) *vs.* control siRNA (Control). Red indicates up-regulation and blue down-regulation. n = 3 samples per group. DESeq2 Wald test was performed for statistical analysis. Genes with a false discovery rate (FDR) adjusted P-values of less than 0.05 were considered as DEGs. P-values were adjusted by using Benjamini-Hochberg (BH) method.

**Figure 3:**
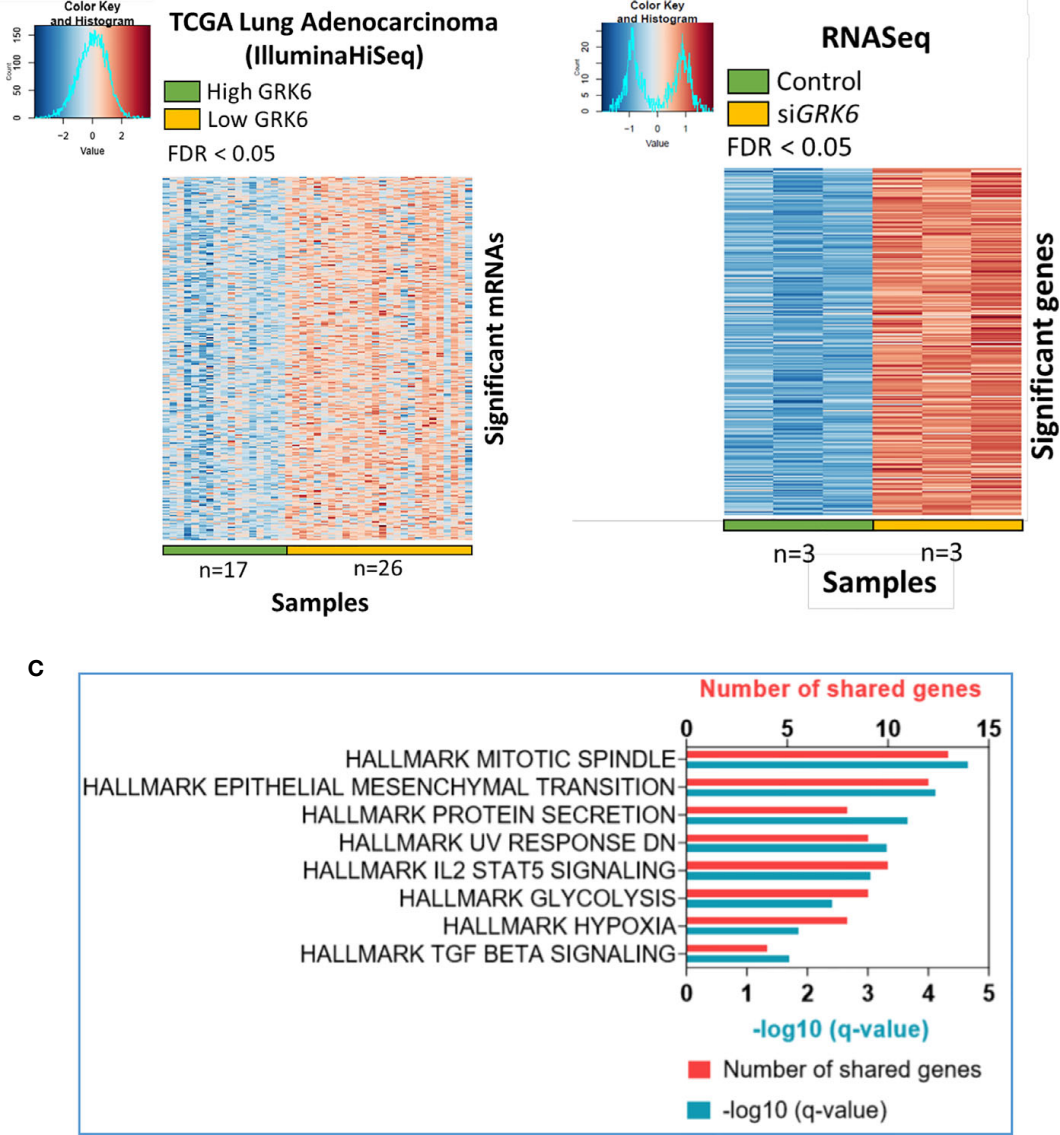
Candidate pathways enriched upon *GRK6* inhibition are identified by TCGA analysis coupled to RNA sequencing. **(A)** Heat-map showing genes that are over-expressed in lung adenocarcinoma samples with low GRK6 (*n* = 26) compared to those with high GRK6 (*n* = 17) from TCGA analysis. Red indicates up-regulation and blue down-regulation. Genes with false discovery rate (FDR) adjusted P-values less than 0.05 were considered as DEGs. P-values were adjusted by using Benjamini-Hochberg (BH) method. **(B)** Heat-map showing DEGs (differentially expressed genes) that are over-expressed in ATII cells transfected with siRNA against *GRK6* (si*GRK6*) *vs.* control siRNA. Red indicates up-regulation and blue down-regulation. *n* = 3 samples per group. DESeq2 Wald test was performed for statistical analysis. Genes with FDR adjusted P-values less than 0.05 were considered as DEGs. P-values were adjusted by using Benjamini-Hochberg (BH) method. **(C)** Pathways enriched upon *GRK6* inhibition are visualised on a bar chart, showing number of shared genes and -Log_10_ (q value).

To understand the role of GRK6 in lung adenocarcinoma, we performed TCGA analysis coupled with the RNA-seq data described above. As shown in **Figure 2A**, correlation analysis was performed in the TCGA lung adenocarcinoma (LUAD) (IlluminaHiseq) dataset; samples were separated into high *vs.* low *GRK6* expression based on an unsupervised hierarchical clustering (**Supplementary Figure 2**). We identified 2,345 genes as differentially expressed in the high *vs.* low *GRK6* samples in the TCGA dataset (**Figure 2B**). A total of 7,116 genes were differentially expressed in ATII cells transfected with control siRNA or siRNA against *GRK6* (si*GRK6*) in RNA-seq, among which 3,430 up-regulated (**Figure 2C**). By cross-referencing the results from the TCGA analysis with the RNA-seq analysis, we identified 274 candidate genes, which were highly expressed in low *GRK6* samples in the TCGA dataset (**Figure 3A**; **Supplementary Table 5**) and in si*GRK6* samples in the RNA-seq analysis (**Figure 3B**; **Supplementary Table 6**).

Metascape (https://metascape.org/gp/index.html#/main/step1) was used to investigate whether these genes were enriched in certain cellular pathways. We found that several Hallmark pathways, including mitotic spindle, epithelial mesenchymal transition (EMT), protein secretion, IL2 (interleukin 2) STAT5 (signal transducer and activator of transcription 5) signalling, glycolysis, hypoxia and TGFβ signalling, were enriched upon GRK6 inhibition in lung adenocarcinoma (**Figure 3C**; **Table 1**).

**Table 1:** List of pathways enriched upon *GRK6* inhibition.

	Number of shared genes	-Log_10_ (q-value)	Genes
**HALLMARK MITOTIC SPINDLE**	13	4.653	*APC, ARHGAP5, NOTCH2, RFC1, ROCK1, TIAM1, TRIO, ARHGAP29, RASAL2, ARHGEF12, SUN2, DYNLL2, PPP4R2*
**HALLMARK EPITHELIAL MESENCHYMAL TRANSITION**	12	4.122	*CALU, CD44, CD59, DPYSL3, FBN2, FN1, ITGAV, NOTCH2, PTX3, SDC1, TGFBI, SLIT2*
**HALLMARK PROTEIN SECRETION**	8	3.664	*CLCN3, GOLGA4, IGF2R, PAM, RPS6KA3, ZW10, SCRN1, STX12*
**HALLMARK UV RESPONSE DN**	9	3.321	*RUNX1, LTBP1, NOTCH2, ATXN1, NRP1, MAGI2, NR1D2, SIPA1L1, MIOS*
**HALLMARK IL2 STAT5 SIGNALING**	10	3.042	*CD44, IGF2R, ITGAV, PRNP, TIAM1, NRP1, DENND5A, TWSG1, RRAGD, SPRED2*
**HALLMARK GLYCOLYSIS**	9	2.418	*CD44, ENO1, IL13RA1, PAM, SDC1, TGFBI, P4HA2, HS2ST1, RRAGD*
**HALLMARK HYPOXIA**	8	1.847	*ENO1, GBE1, PAM, PFKFB3, TGFBI, P4HA2, KDM3A, RRAGD*
**HALLMARK TGF BETA SIGNALING**	4	1.701	*ACVR1, APC, SLC20A1, NOG*

### *GRK6* Inhibition Induces Hypoxia-Inducible Factor (HIF) Activity in the Lungs

**Figure 4:**
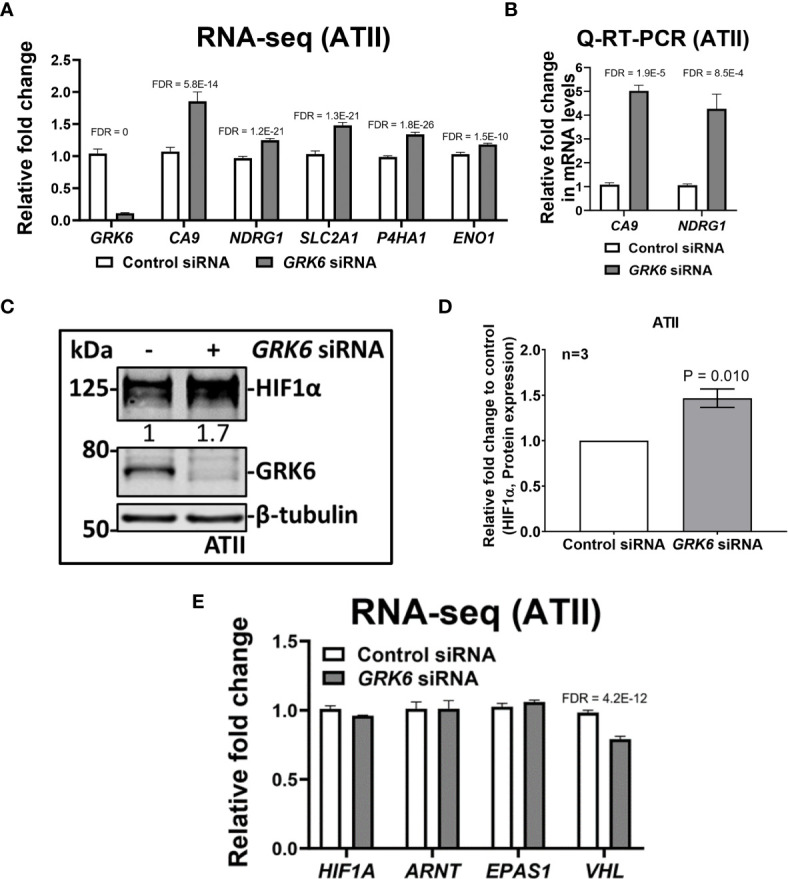
*GRK6* depletion induces HIF (hypoxia-inducible factors) activity in ATII (alveolar epithelial type II) cells. **(A)** RNA-seq showing relative expressions of *GRK6*, *CA9*, *NDRG1, SLC2A1, P4HA1* and *ENO1* in ATII cells transfected with control or *GRK6* siRNA. Data are mean ± s.d. *n* = 3 samples per group. Multiple t-test was performed for statistical analysis. Genes with false discovery rate (FDR) adjusted P-values less than 0.05 were considered as DEGs. P-values were adjusted by using Benjamini-Hochberg (BH) method. **(B)** Fold change in mRNA levels of *CA9* and *NDRG1* in ATII cells transfected with control or *GRK6* siRNA. *ACTB* (β-actin)-normalised mRNA levels in control cells were used to set the baseline value at unity. Data are mean ± s.d. *n* = 3 samples per group. Multiple t-test was performed for statistical analysis. **(C)** Protein expression of HIF1α and GRK6 in ATII cells transfected with control or *GRK6* siRNA. β-tubulin was used as a loading control. **(D)** Quantification of **(C)**. Graph showing protein level of HIF1α in ATII cell line with indicated transfections. Data are mean ± s.d. *n* = 3 per group. Two tailed, unpaired Student’s *t-test* was performed for statistical analysis. P-value less than 0.05 was considered as statistically significant. **(E)** RNA-seq showing relative expression of *HIF1A*, *ARNT*, *EPAS1* and *VHL* in ATII cells transfected with control or *GRK6* siRNA. Data are mean ± s.d. *n* = 3 samples per group. Multiple t-test was performed for statistical analysis. Genes with FDR adjusted P-values less than 0.05 were considered as DEGs. P-values were adjusted by using Benjamini-Hochberg (BH) method.

One of the Hallmark pathways enriched upon GRK6 inhibition is ‘Hallmark_Hypoxia’ (FDR = 0.014; **Figure 3C**; **Table 1**). In our RNA-seq analysis, knockdown of *GRK6* in ATII cells (**Figure 4A**) led to significant increases in several hypoxia-induced genes, including *CA9* (carbonic anhydrase 9), *NDRG1* (N-Myc downstream-regulated 1), *SLC2A1* (solute carrier family 2 member 1, also known as *GLUT1*, glucose transporter 1), *P4HA1* (prolyl 4-hydroxylase subunit alpha 1) and *ENO1* (enolase 1) (28) (**Figure 4A**). A significant increase in the mRNA levels of *CA9* (*P* < 0.0001) and *NDRG1* (*P* < 0.001) were confirmed with Q-RT-PCR (**Figure 4B**). In addition, the protein level of HIF1α, a key regulator of the cellular response to hypoxia (29), was significantly increased upon *GRK6* depletion in the ATII cells as shown by western blot (**Figures 4C, D**; *P* < 0.01). To check how GRK6 may regulate HIF activity, the mRNA levels of HIF1α (*HIF1A*), HIF2α (*EPAS1*), HIF1β (*ARNT*) and *VHL* (Von Hippel-Lindau) were screened in the RNA-seq dataset. No changes in the expression levels of *HIF1A*, *EPAS1* and *ARNT* were observed (**Figure 4E**; P > 0.05), while the *VHL* mRNA level was decreased upon GRK6 inhibition in ATII cells (**Figure 4E**; P < 0.001). These findings suggest that GRK6 inhibition induces HIF activity in the lungs potentially by regulating VHL, which functions as a master regulator of HIF activity by targeting the HIFα subunit for degradation (30–33).

**Figure 5:**
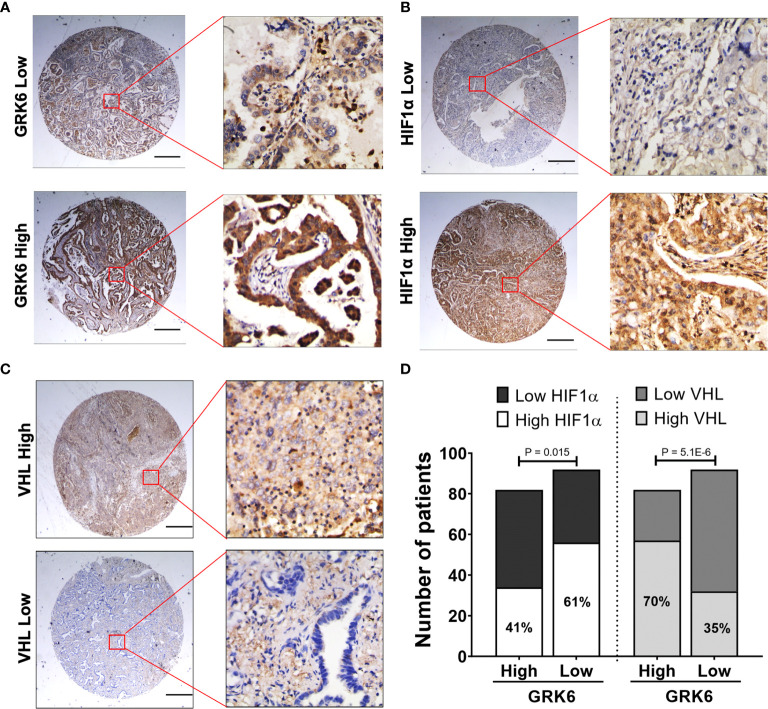
GRK6 expression levels negatively and positively correlate with HIF1α and VHL expressions in lung adenocarcinoma, respectively. **(A)** Representative GRK6 staining pattern (low or high GRK6) in lung adenocarcinoma tissue microarray cores. Scale bar: 500 μm. **(B)** Representative HIF1α staining pattern (low or high HIF1α) in lung adenocarcinoma tissue microarray cores. Scale bar: 500 μm. **(C)** Representative VHL staining pattern (low or high VHL) in lung adenocarcinoma tissue microarray cores. Scale bar: 500 μm. **(D)** Graph showing the number and percentage of lung adenocarcinoma patients with low/high HIF1α or low/high VHL in high *vs.* low GRK6 group. High GRK6 *n* = 82. Low GRK6 *n* = 92. Fisher’s exact test was performed for statistical analysis. P-values less than 0.05 were considered as statistically significant.

To further validate the *in vitro* findings, the correlation between GRK6 expression and HIF1α levels or GRK6 expression and VHL levels were analysed in lung adenocarcinoma samples using tissue microarrays (**Figure 5**). Representative images of low and high expression of GRK6, HIF1α or VHL in lung adenocarcinoma samples are shown in **Figures 5A–C**, respectively. Importantly, the percentage of patients with high HIF1α expression (61%) in the low GRK6 group was significantly higher than in the high GRK6 group (41%) (**Figure 5D**; *P* < 0.05), while patients with low GRK6 tended to have a low level of VHL compared to those within high GRK6 group (**Figure 5D**; *P* < 0.0001).

## Discussion

Lung cancer is the most prevalent and the leading cause of cancer death (34). Adenocarcinoma is the most common type of lung cancer, in both smokers and non-smokers, in females and males, and represents 40% of the lung cancer cases (35). Lung adenocarcinoma progresses from the small airway; one of the most abundant cell types present here are alveolar type II epithelial cells, which secrete mucus and other substances (36). Lung adenocarcinoma is one of the most aggressive cancers and the survival rate of patients is short after diagnosis with an overall survival rate of less than 5 years (35). The major challenge for lung adenocarcinoma is its resistance to conventional radiotherapies and chemotherapies (35).

Hypoxia is one of the typical features of the tumour microenvironment that increases the aggressiveness of different tumours such as lung cancer (37), colorectal cancer (38), hepatocellular carcinoma (39) and oesophageal squamous cell carcinoma (40). Hypoxic conditions lead to the activation of various transcription factors, such as HIF1; and the activation of downstream signalling pathways that regulate cell death, motility and proliferation (41). HIF1 is a heterodimeric transcription factor, capable of controlling the cellular adaptive response to hypoxia and has two subunits; HIF1α and HIF1β (42, 43). Cellular oxygen concentration regulates the protein expression of HIF1α so is a key factor for cellular adaptive response to hypoxia (43). HIF activities can also be up-regulated by other mechanisms (44, 45).

G protein-coupled receptor kinases (GRKs) are a family of kinases which can desensitize G protein-coupled receptors (GPCRs) homologous (1). GRK6 is of the members of the GRK family (10–12) and we previously showed that GRK6 is down-regulated in lung adenocarcinoma, which is associated with malignant tumour progression (16, 17), by an unknown mechanism.

To identify global transcriptomic changes in ATII cells upon *GRK6* depletion, RNA-seq coupled with siRNA-mediated depletion of *GRK6* was performed in ATII cells. We identified 3,430 up-regulated and 3,686 down-regulated DEGs. GO functional analysis with DEGs demonstrated that DEGs are mainly enriched in mRNA metabolism, ribonucleoprotein complex biogenesis, and regulation of cellular response to stress. To understand the role of GRK6 in lung adenocarcinoma, analysis of TCGA data was coupled with the RNA-seq data, described above. Pathway analysis suggested that one of the Hallmark pathways enriched upon GRK6 inhibition is ‘Hallmark_Hypoxia’. We demonstrated that *GRK6* depletion induces HIF1α expression and activity in ATII cells. The findings were further confirmed in lung adenocarcinoma samples, in which GRK6 expressions negatively correlate with HIF1α protein levels. Mechanistically, the impact of GRK6 on HIF activity could be achieved *via* regulation of VHL levels, which is a master regulator of HIF activity by targeting the prolyl-hydroxylated HIF1α subunit for ubiquitylation and rapid proteasomal degradation (30–33). This study provides evidence that GRK6 inhibition causes a decrease in VHL expression, leading to HIFα stabilisation with increased activity in lung adenocarcinoma, although the underlying mechanism merits further investigation.

Earlier reports suggest that hypoxia regulates mRNA translation (46). RNA-binding proteins (heterogeneous nuclear ribonucleoproteins) have a role in post-transcriptional gene regulation under hypoxic conditions and are associated with hypoxia-induced transcripts that regulate encoded protein levels (47). Hypoxia can affect tumour cells; by acting as a stressor and inhibiting cell growth or inducing cell death. Alternatively, it can act by contributing to cancer progression and resistance to treatments, leading to hypoxia-induced genomic and proteomic changes in the cancer cells (48, 49).

We previously demonstrated that cell migration and invasion in lung epithelial cells is induced upon *GRK6* knockdown (17). In addition to the hypoxia, this analysis showed EMT is also enriched upon *GRK6* inhibition, which can explain our previous findings (17). The hypoxic tumour microenvironment can regulate EMT (50, 51). EMT is a biological process and the cell polarity and cell-cell adhesion of epithelial cells are lost and in turn become mesenchymal cells, which have migratory and invasive features (52). In a similar manner to our findings (17), previous studies in medulloblastoma (53) and Lewis lung carcinoma (2) show that when *GRK6* was downregulated, migration and metastasis were increased. Consistently, it has been found that hypoxia-related genes *CA9*, *NDRG1*, *SLC2A1*, *P4HA1* and *ENO1* induced EMT in hepatocellular carcinoma (54), bladder cancer (55), laryngeal cancer (56) and gastric cancer (57, 58), respectively. Our study showed an increase of hypoxia-induced gene expression and HIF1α expression in *GRK6* knockdown cells, this suggests that *GRK6* knockdown may induce EMT in lung adenocarcinoma.

In summary, this study shows that *GRK6* is involved in different disease-related pathological features; mRNA metabolism, ribonucleoprotein complex biogenesis, regulation of cellular response to stress, as well as EMT and hypoxia. Targeting the HIF pathway may provide new strategies for therapy in *GRK6*-depleted lung adenocarcinoma patients.

## Data Availability Statement

The datasets presented in this study can be found in online repositories. The names of the repository/repositories and accession number(s) can be found below: https://www.ncbi.nlm.nih.gov/geo/, GSE164921.

## Ethics Statement

This study was approved by the Ethics Committee of the Affiliated Hospital of Nantong University (No. 2018-L068). The patients/participants provided their written informed consent to participate in this study.

## Author Contributions

Conceptualization and supervision: YW, YL, and XL. Investigation: SY, AE, YZ, LY, CH, JC, YG, and HS. Formal analysis: SY, AE, YZ, HS, and YW. Writing: AE, SY, YZ, LY, and CH with support from YW and RE. Funding acquisition: YW, YL, and SY. All authors contributed to the article and approved the submitted version.

## Funding

This project was funded by grants from Medical Research Council (UK) [MR/S025480/1], Jiangsu Post-doctoral Foundation Research Project, China [No. 2019Z142], Key Talents of Medical Science in Jiangsu Province, China [No. QNRC2016682], and Science and Technology Project of Nantong [No. JCZ18130]. AE was supported by the Wessex Medical Trust. YZ was supported by an Institute for Life Sciences (University of Southampton) PhD Studentship. CH was supported by Gerald Kerkut Charitable Trust and University of Southampton Central VC Scholarship Scheme.

## Conflict of Interest

The authors declare that the research was conducted in the absence of any commercial or financial relationships that could be construed as a potential conflict of interest.

## References

[1] Vroon A Heijnen CJ Kavelaars A. GRKs and Arrestins: Regulators of Migration and Inflammation. J Leukocyte Biol (2006) 80(6):1214–21. 10.1189/jlb.0606373 16943386

[2] Raghuwanshi SK Smith N Rivers EJ Thomas AJ Sutton N Hu Y. GRK6 Deficiency Promotes Angiogenesis, Tumor Progression and Metastasis. J Immunol (2013) 190(10):5329–36. 10.4049/jimmunol.1202058.GRK6 PMC3646980 23589623

[3] Bouvier M Hausdorff WP De Blasi A O’Dowd BF Kobilka BK Caron MG. Removal of Phosphorylation Sites From the β2-Adrenergic Receptor Delays Onset of Agonist-Promoted Desensitization. Nature (1988) 333(6170):370–3. 10.1038/333370a0 2836733

[4] Balabanian K Lagane B Pablos JL Laurent L Planchenault T Verola O. WHIM Syndromes With Different Genetic Anomalies are Accounted for by Impaired CXCR4 Desensitization to CXCL12. Blood (2005) 105(6):2449–57. 10.1182/blood-2004-06-2289 15536153

[5] Wang WCH Mihlbachler KA Brunnett AC Liggett SB. Targeted Transgenesis Reveals Discrete Attenuator Functions of GRK and PKA in Airway β2-Adrenergic Receptor Physiologic Signaling. Proc Natl Acad Sci USA (2009) 106(35):15007–12. 10.1073/pnas.0906034106 PMC2736457 19706446

[6] Rockman HA Chien KR Choi DJU Iaccarino G Hunter JJ John Ross JR. Expression of a β-Adrenergic Receptor Kinase 1 Inhibitor Prevents the Development of Myocardial Failure in Gene-Targeted Mice. Proc Natl Acad Sci USA (1998) 95(12):7000–5. 10.1073/pnas.95.12.7000 PMC22717 9618528

[7] Gainetdinov RR Bohn LM Sotnikova TD Cyr M Laakso A Macrae AD. Dopaminergic Supersensitivity in G Protein-Coupled Receptor Kinase 6-Deficient Mice. Neuron (2003) 38(2):291–303. 10.1016/S0896-6273(03)00192-2 12718862

[8] Barak LS Oakley RH Laporte SA Caron MG. Constitutive Arrestin-Mediated Desensitization of a Human Vasopressin Receptor Mutant Associated With Nephrogenic Diabetes Insipidus. Proc Natl Acad Sci USA (2001) 98(1):93–8. 10.1073/pnas.98.1.93 PMC14550 11134505

[9] Yu S Sun L Jiao Y Lee LTO. The Role of G Protein-Coupled Receptor Kinases in Cancer. Int J Biol Sci (2018) 14(2):189–203. 10.7150/ijbs.22896 29483837 PMC5821040

[10] Ahmed MR Berthet A Bychkov E Porras G Li Q Bioulac BH. Lentiviral Overexpression of GRK6 Alleviates L-dopa-induced Dyskinesia in Experimental Parkinson’s Disease. Sci Trans Med (2010) 2(28):28ra28. 10.1126/scitranslmed.3000664 PMC2933751 20410529

[11] Tiedemann RE Zhu YX Schmidt J Yin H Shi CX Que Q. Kinome-Wide RNAi Studies in Human Multiple Myeloma Identify Vulnerable Kinase Targets, Including a Lymphoid-Restricted Kinase, GRK6. Blood (2010) 115(8):1594–604. 10.1182/blood-2009-09-243980 PMC2830764 19996089

[12] Willets JM John Challiss RA Nahorski SR. Endogenous G Protein-Coupled Receptor Kinase 6 Regulates M3 Muscarinic Acetylcholine Receptor Phosphorylation and Desensitization in Human SH-SY5Y Neuroblastoma Cells. J Biol Chem (2002) 277(18):15523–9. 10.1074/jbc.M111217200 11856737

[13] Li YP. GRK6 Expression in Patients With Hepatocellular Carcinoma. Asian Pacific J Trop Med (2013) 6(3):220–3. 10.1016/S1995-7645(13)60027-9 23375037

[14] Tao R Li Q Gao X Ma L. Overexpression of GRK6 Associates With the Progression and Prognosis of Colorectal Carcinoma. Oncol Lett (2018) 15(4):5879–86. 10.3892/ol.2018.8030 PMC5840531 29552218

[15] Qiu X Chen J Zhang Z You Y Wang Z. Aberrant GRK6 Promoter Methylation is Associated With Poor Prognosis in Hypopharyngeal Squamous Cell Carcinoma. Oncol Rep (2016) 35(2):1027–33. 10.3892/or.2015.4469 26718636

[16] Yao S Zhong L Liu J Feng J Bian T Zhang Q. Prognostic Value of Decreased GRK6 Expression in Lung Adenocarcinoma. J Cancer Res Clin Oncol (2016) 142(12):2541–9. 10.1007/s00432-016-2244-y PMC11818988 27601164

[17] Yao S Wu D Chen J Wang P Lv X Huang J. Hypermethylation of the G Protein-Coupled Receptor Kinase 6 (GRK6) Promoter Inhibits Binding of C/EBPα, and GRK6 Knockdown Promotes Cell Migration and Invasion in Lung Adenocarcinoma Cells. FEBS Open Bio (2019) 9(4):605–17. 10.1002/2211-5463.12606 PMC6443861 30984536

[18] Hill C Li J Liu D Conforti F Brereton CJ Yao L. Autophagy Inhibition-Mediated Epithelial–Mesenchymal Transition Augments Local Myofibroblast Differentiation in Pulmonary Fibrosis. Cell Death Dis (2019) 10(8):591. 10.1038/s41419-019-1820-x 31391462 PMC6685977

[19] Molina-Arcas M Hancock DC Sheridan C Kumar MS Downward J. Coordinate Direct Input of Both KRAS and IGF1 Receptor to Activation of PI3 Kinase in KRAS -Mutant Lung Cancer. Cancer Discovery (2013) 3(5):548–63. 10.1158/2159-8290.CD-12-0446 PMC3650991 23454899

[20] Coelho MA de Carné Trécesson S Rana S Zecchin D Moore C Molina-Arcas M. Oncogenic RAS Signaling Promotes Tumor Immunoresistance by Stabilizing PD-L1 mRNA. Immunity (2017) 47(6):1083–99.e6. 10.1016/j.immuni.2017.11.016 29246442 PMC5746170

[21] Yao L Conforti F Hill C Bell J Drawater L Li J. Paracrine Signalling During ZEB1-Mediated Epithelial–Mesenchymal Transition Augments Local Myofibroblast Differentiation in Lung Fibrosis. Cell Death Differentiation (2019) 26(5):943–57. 10.1038/s41418-018-0175-7 PMC6252080 30050057

[22] Ewels P Magnusson M Lundin S Käller M. MultiQC: Summarize Analysis Results for Multiple Tools and Samples in a Single Report. Bioinformatics (2016) 32(19):3047–8. 10.1093/bioinformatics/btw354 PMC5039924 27312411

[23] Kim D Langmead B Salzberg SL. Hisat: A Fast Spliced Aligner With Low Memory Requirements. Nat Methods (2015) 12(4):357–60. 10.1038/nmeth.3317 PMC4655817 25751142

[24] Li H Handsaker B Wysoker A Fennell T Ruan J Homer N. The Sequence Alignment/Map Format and Samtools. Bioinformatics (2009) 25(16):2078–9. 10.1093/bioinformatics/btp352 PMC2723002 19505943

[25] Liao Y Smyth GK Shi W. Featurecounts: An Efficient General Purpose Program for Assigning Sequence Reads to Genomic Features. Bioinformatics (2014) 30(7):923–30. 10.1093/bioinformatics/btt656 24227677

[26] Love MI Huber W Anders S. Moderated Estimation of Fold Change and Dispersion for RNA-seq Data With DESeq2. Genome Biol (2014) 15(12):550. 10.1186/s13059-014-0550-8 25516281 PMC4302049

[27] Sun R Wang X Zhu H Mei H Wang W Zhang S. Prognostic Value of LAMP3 and TP53 Overexpression in Benign and Malignant Gastrointestinal Tissues. Oncotarget (2014) 5(23):12398–409. 10.18632/oncotarget.2643 PMC4322976 25362357

[28] Buffa FM Harris AL West CM Miller CJ. Large Meta-Analysis of Multiple Cancers Reveals a Common, Compact and Highly Prognostic Hypoxia Metagene. Br J Cancer (2010) 102(2):428–35. 10.1038/sj.bjc.6605450 PMC2816644 20087356

[29] Kaelin WG Ratcliffe PJ. Oxygen Sensing by Metazoans: The Central Role of the HIF Hydroxylase Pathway. Mol Cell (2008) 30(4):393–402. 10.1016/j.molcel.2008.04.009 18498744

[30] Cockman ME Masson N Mole DR Jaakkola P Chang GW Clifford SC. Hypoxia Inducible Factor-α Binding and Ubiquitylation by the Von Hippel-Lindau Tumor Suppressor Protein. J Biol Chem (2000) 275(33):25733–41. 10.1074/jbc.M002740200 10823831

[31] Ohh M Park CW Ivan M Hoffman MA Kim TY Huang LE. Ubiquitination of Hypoxia-Inducible Factor Requires Direct Binding to the β-Domain of the Von Hippel - Lindau Protein. Nat Cell Biol (2000) 2(7):423–7. 10.1038/35017054 10878807

[32] Schofield CJ Ratcliffe PJ. Oxygen Sensing by HIF Hydroxylases. Nat Rev Mol Cell Biol (2004) 5(5):343–54. 10.1038/nrm1366 15122348

[33] Ratcliffe PJ. Oxygen Sensing and Hypoxia Signalling Pathways in Animals: The Implications of Physiology for Cancer. J Physiol (2013) 591(8):2027–42. 10.1113/jphysiol.2013.251470 PMC3634517 23401619

[34] Bray F Ferlay J Soerjomataram I Siegel RL Torre LA Jemal A. Global Cancer Statistics 2018: GLOBOCAN Estimates of Incidence and Mortality Worldwide for 36 Cancers in 185 Countries. CA: Cancer J Clin (2018) 68(6):394–424. 10.3322/caac.21492 30207593

[35] Denisenko TV Budkevich IN Zhivotovsky B. Cell Death-Based Treatment of Lung Adenocarcinoma. Cell Death Dis (2018) 9(2):117. 10.1038/s41419-017-0063-y 29371589 PMC5833343

[36] Noguchi M Morikawa A Kawasaki M Matsuno Y Yamada T Hirohashi S. Small Adenocarcinoma of the Lung. Histologic Characteristics and Prognosis. Cancer (1995) 75(12):2844–52. 10.1002/1097-0142(19950615)75:12<2844::aid-cncr2820751209>3.0.co;2-# 7773933

[37] Le QT Chen E Salim A Cao H Kong CS Whyte R. An Evaluation of Tumor Oxygenation and Gene Expression in Patients With Early Stage non-Small Cell Lung Cancers. Clin Cancer Res (2006) 12(5):1507–14. 10.1158/1078-0432.CCR-05-2049 16533775

[38] Qureshi-Baig K Kuhn D Viry E Pozdeev VI Schmitz M Rodriguez F. Hypoxia-Induced Autophagy Drives Colorectal Cancer Initiation and Progression by Activating the PRKC/PKC-EZR (ezrin) Pathway. Autophagy (2020) 16(8):1436–52. 10.1080/15548627.2019.1687213 PMC7469473 31775562

[39] Kung-Chun Chiu D Pui-Wah Tse A Law CT Ming-Jing Xu I Lee D Chen M. Hypoxia Regulates the Mitochondrial Activity of Hepatocellular Carcinoma Cells Through HIF/HEY1/PINK1 Pathway. Cell Death Dis (2019) 10(12):934. 10.1038/s41419-019-2155-3 31819034 PMC6901483

[40] Zhang Q Zhang J Fu Z Dong L Tang Y Xu C. Hypoxia-Induced microRNA-10b-3p Promotes Esophageal Squamous Cell Carcinoma Growth and Metastasis by Targeting TSGA10. Aging (2019) 11(22):10374–84. 10.18632/aging.102462 PMC6914416 31772141

[41] Semenza GL. Hypoxia-Inducible Factors: Mediators of Cancer Progression and Targets for Cancer Therapy. Trends Pharmacol Sci (2012) 33(4):207–14. 10.1016/j.tips.2012.01.005 PMC3437546 22398146

[42] Semenza GL. Hypoxia, Clonal Selection, and the Role of HIF-1 in Tumor Progression. Crit Rev Biochem Mol Biol (2000) 35(2):71–103. 10.1080/10409230091169186 10821478

[43] Jiang BH Semenza GL Bauer C Marti HH. Hypoxia-Inducible Factor 1 Levels Vary Exponentially Over a Physiologically Relevant Range of O2 Tension. Am J Physiol - Cell Physiol (1996) 271(4):C1172–80. 10.1152/ajpcell.1996.271.4.c1172 8897823

[44] Zhang WC Wells JM Chow K-H Huang H Yuan M Saxena T. miR-147b-Mediated TCA Cycle Dysfunction and Pseudohypoxia Initiate Drug Tolerance to EGFR Inhibitors in Lung Adenocarcinoma. Nat Metab (2019) 1(4):460–74. 10.1038/s42255-019-0052-9 PMC6750230 31535082

[45] Zhao F Malm SW Hinchman AN Li H Beeks CG Klimecki WT. Arsenite-Induced Pseudo-Hypoxia Results in Loss of Anchorage-Dependent Growth in BEAS-2B Pulmonary Epithelial Cells. PloS One (2014) 9(12):e114549. 10.1371/journal.pone.0114549 25513814 PMC4267735

[46] Liu L Cash TP Jones RG Keith B Thompson CB Simon MC. Hypoxia-Induced Energy Stress Regulates mRNA Translation and Cell Growth. Mol Cell (2006) 21(4):521–31. 10.1016/j.molcel.2006.01.010 PMC3153113 16483933

[47] Yang R Weber DJ Carrier F. Post-Transcriptional Regulation of Thioredoxin by the Stress Inducible Heterogenous Ribonucleoprotein A18. Nucleic Acids Res (2006) 34(4):1224–36. 10.1093/nar/gkj519 PMC1388095 16513844

[48] Höckel M Vaupel P. Tumor Hypoxia: Definitions and Current Clinical, Biologic, and Molecular Aspects. J Natl Cancer Institute (2001) 93(4):266–76. 10.1093/jnci/93.4.266 11181773

[49] Vaupel P Thews O Hoeckel M. Treatment Resistance of Solid Tumors: Role of Hypoxia and Anemia. Med Oncol (2001) 18(4):243–59. 10.1385/MO:18:4:243 11918451

[50] Joseph JP Harishankar MK Pillai AA Devi A. Hypoxia Induced EMT: A Review on the Mechanism of Tumor Progression and Metastasis in OSCC. Oral Oncol (2018) 80(1):23–32. 10.1016/j.oraloncology.2018.03.004 29706185

[51] Wei L Sun JJ Cui YC Jiang SL Wang XW Lv LY. Twist may be Associated With Invasion and Metastasis of Hypoxic NSCLC Cells. Tumor Biol (2016) 37(7):9979–87. 10.1007/s13277-016-4896-2 26819207

[52] Polyak K Weinberg RA. Transitions Between Epithelial and Mesenchymal States: Acquisition of Malignant and Stem Cell Traits. Nat Rev Cancer (2009) 9(4):265–73. 10.1038/nrc2620 19262571

[53] Yuan L Zhang H Liu J Rubin JB Cho YJ Shu HK. Growth Factor Receptor-Src-mediated Suppression of GRK6 Dysregulates CXCR4 Signaling and Promotes Medulloblastoma Migration. Mol Cancer (2013) 12(1):18. 10.1186/1476-4598-12-18 23497290 PMC3599655

[54] Hyuga S Wada H Eguchi H Otsuru T Iwgami Y Yamada D. Expression of Carbonic Anhydrase IX is Associated With Poor Prognosis Through Regulation of the Epithelial-Mesenchymal Transition in Hepatocellular Carcinoma. Int J Oncol (2017) 51(4):1179–90. 10.3892/ijo.2017.4098 28849188

[55] Li A Zhu X Wang C Yang S Qiao Y Qiao R. Upregulation of NDRG1 Predicts Poor Outcome and Facilitates Disease Progression by Influencing the EMT Process in Bladder Cancer. Sci Rep (2019) 9(1):5166. 10.1038/s41598-019-41660-w 30914736 PMC6435802

[56] Starska K Forma E Jóźwiak P Bryś M Lewy-Trenda I Brzezińska-Błaszczyk E. Gene and Protein Expression of Glucose Transporter 1 and Glucose Transporter 3 in Human Laryngeal Cancer—the Relationship With Regulatory Hypoxia-Inducible Factor-1α Expression, Tumor Invasiveness, and Patient Prognosis. Tumor Biol (2015) 36(4):2309–21. 10.1007/s13277-014-2838-4 PMC4428538 25412955

[57] Zhang J Guo S Wu Y Zheng ZC Wang Y Zhao Y. P4HB, a Novel Hypoxia Target Gene Related to Gastric Cancer Invasion and Metastasis. BioMed Res Int (2019) 2019(1):9749751. 10.1155/2019/9749751 31467922 PMC6699373

[58] Xu X Chen B Zhu S Zhang J He X Cao G. Hyperglycemia Promotes Snail-induced Epithelial-Mesenchymal Transition of Gastric Cancer *Via* Activating ENO1 Expression. Cancer Cell Int (2019) 19(1):344. 10.1186/s12935-019-1075-8 31889896 PMC6924061

