# Supplementary material for: GRK6 Depletion Induces HIF Activity in Lung Adenocarcinoma
Source: Front Oncol. 2021 May 31;11:654812. doi: 10.3389/fonc.2021.654812 (PMC8201516; doi:10.3389/fonc.2021.654812)
Supplement: Supplementary file 1 [file DataSheet_1.pdf]

Supplementary Material

1 Supplementary Tables

1.1 **Supplementary Table 1** Up-regulated DEGs in ATII cells transfected with siRNA against *GRK6 vs.* control siRNA.

1.2 **Supplementary Table 2** Down-regulated DEGs in ATII cells transfected with siRNA against *GRK6 vs.* control siRNA.

1.3 **Supplementary Table 3** GO analysis of up-regulated DEGs in ATII cells transfected with siRNA against *GRK6 vs.* control siRNA**.**

1.4 **Supplementary Table 4** GO analysis of down-regulated DEGs in ATII cells transfected with siRNA against *GRK6 vs.* control siRNA.

1.5 **Supplementary Table 5** List of candidate genes that are over-expressed in lung adenocarcinoma samples with low GRK6 compared to those with high GRK6 from TCGA analysis.

1.6 **Supplementary Table 6** List of candidate genes that are over-expressed in ATII cells transfected with siRNA against *GRK6* (si*GRK6*) *vs.* control siRNA.

2 Supplementary Figures

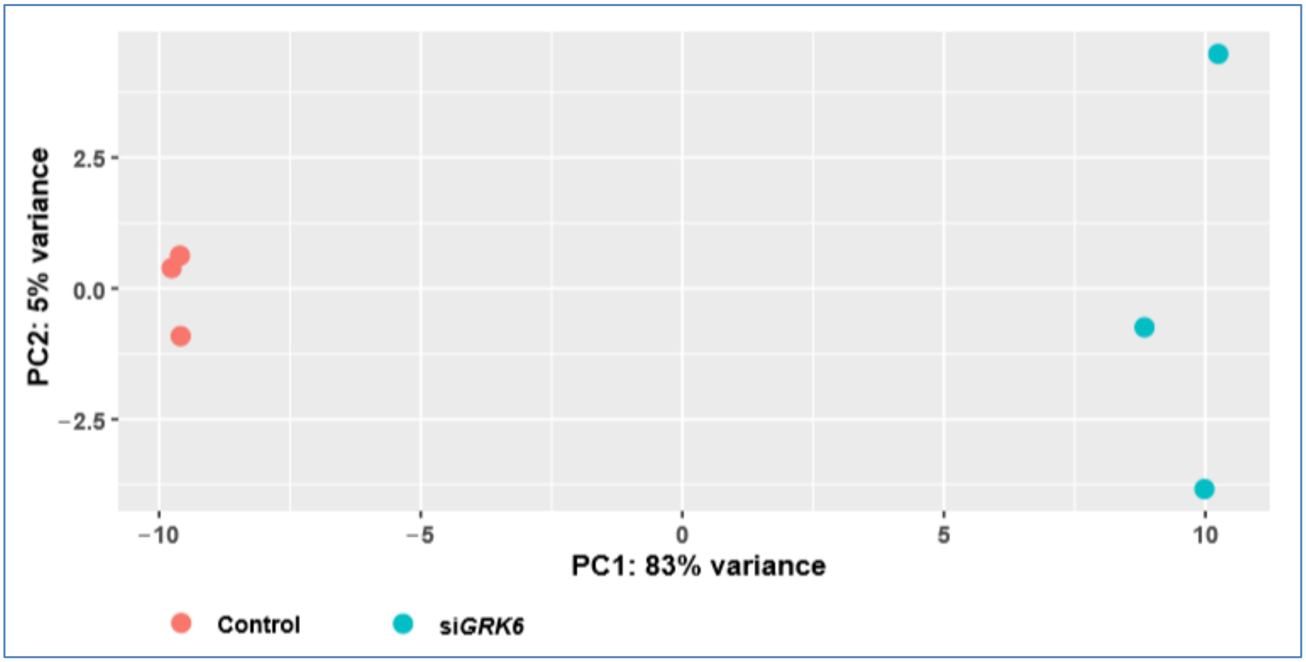

Supplementary Figure 1. Principal component analysis (PCA) between control and si*GRK6* samples from RNA-Seq data. ATII cells were transfected with control siRNA (Control, in red) or siRNA against *GRK6* (si*GRK6*, in blue) for 3 days, followed by RNA-Seq analysis. Each point represents an RNA-Seq sample. Samples that have similar gene expression patterns are clustered together.

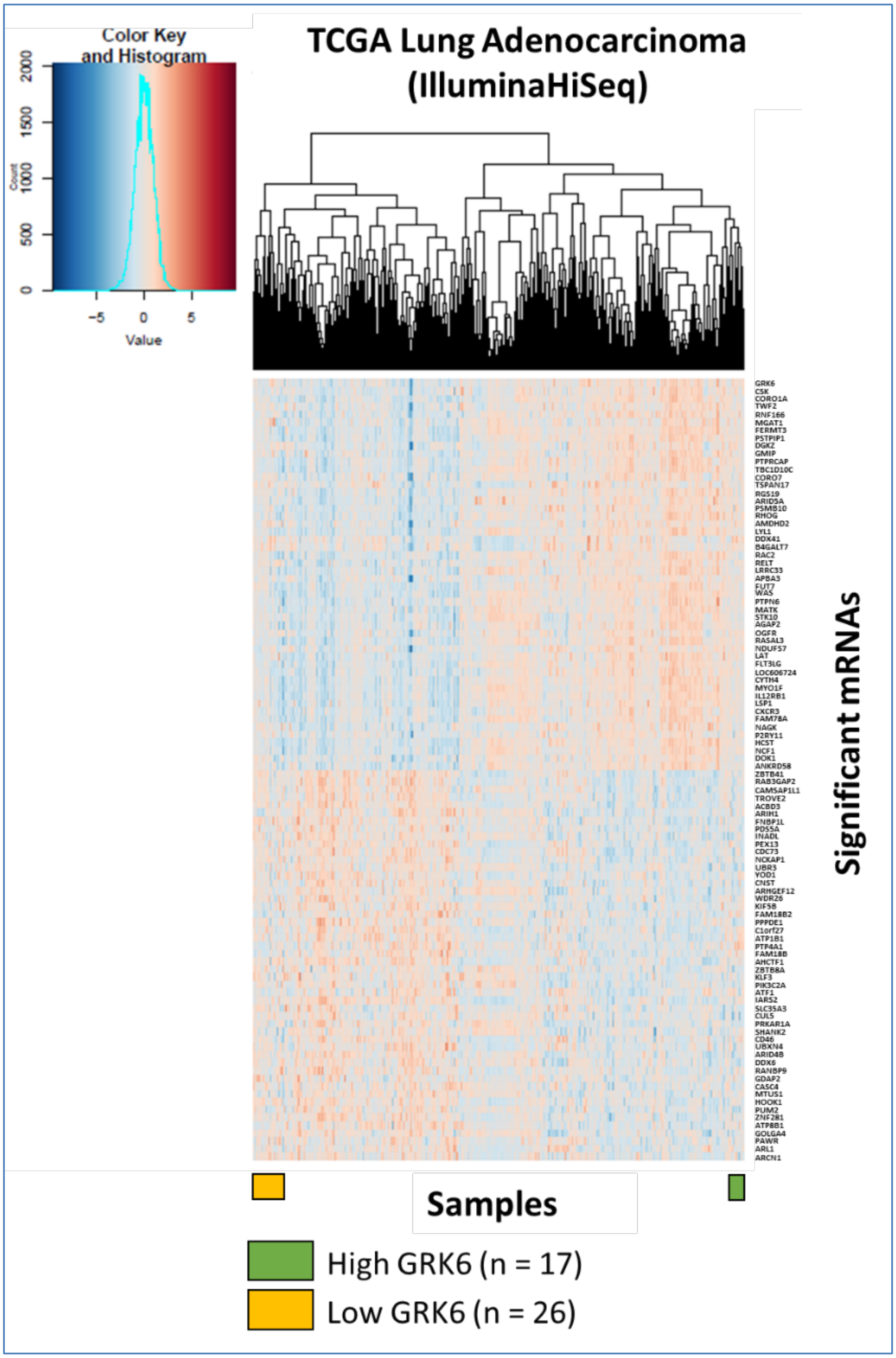

Supplementary Figure 2. Lung adenocarcinoma samples with either low or high GRK6 are identified in TCGA by unsupervised hierarchical clustering. Heat-map showing genes that are positively or negatively correlated with GRK6 expression levels in TCGA lung adenocarcinoma samples. A total of 17 samples were identified as high GRK6 (green box) and 26 as low GRK6 (yellow box). Red in heatmap indicates up-regulation and blue down-regulation.
